# Impact of CA9 expression in the diagnosis of lymph-node metastases in non-small cell lung cancer based on [^18^F]FDG PET/CT

**DOI:** 10.1371/journal.pone.0312846

**Published:** 2024-10-29

**Authors:** Satoshi Suzuki, Masakazu Yashiro, Nobuhiro Izumi, Takuma Tsukioka, Hidetoshi Inoue, Kantaro Hara, Ryuichi Ito, Takuya Tanimura, Noritoshi Nishiyama

**Affiliations:** 1 Department of Thoracic Surgery, Osaka Metropolitan University Graduate School of Medicine, Osaka, Japan; 2 Molecular Oncology and Therapeutics, Osaka Metropolitan University Graduate School of Medicine, Osaka, Japan; 3 Department of Thoracic Surgery, Kansai Rosai Hospital, Hyogo, Japan; Spedali Civili of Brescia, University of Brescia, ITALY

## Abstract

**Background:**

Lung cancer is the leading cause of the global cancer incidence and mortality. It is important to obtain an accurate diagnosis of lymph-node metastasis before surgery to select the therapeutic strategy for non-small cell lung cancer (NSCLC) patients. Carbonic anhydrase 9 (CA9) is considered a marker of hypoxia and it has reported that CA9 is associated with tumor invasion and metastasis. In this study, the correlation between the CA9 expression for lymph-node metastases in NSCLC and [^18^F]FDG PET/CT results was investigated in order to clarify the efficacy of [^18^F]FDG PET/CT for detecting lymph-node metastases of NSCLC patients.

**Methods:**

Among the 564 patients who underwent surgical treatment for NSCLC between 2010 and 2016 at our hospital, a total of 338 patients who underwent preoperative [^18^F]FDG PET/CT were included in this study. CA9 expression was evaluated by immunochemistry. A lymph node with maximum standardized uptake value (SUVmax) ≥2.5 on [^18^F]FDG PET/CT was preoperatively defined as a metastatic lymph node.

**Result:**

CA9 positivity was detected in 122 patients; the other 216 patients were CA9-negative. The CA9-positive NSCLC cases significantly associated with pleural invasion (*p* = 0.0063), pT-factor (*p* = 0.0080), pN-factor (*p* = 0.036) and pStage (*p* = 0.043). CA9-positive patients presented significantly poorer survival rate for OS than that of the CA9-negative patients (*p* = 0.0024). In the multivariable analysis, histological SCC and CA9 positivity were independent poor-prognosis factors for OS. For the total patient population, the sensitivity and specificity of [^18^F]FDG PET/CT for lymph-node metastases were 54% and 89%, respectively. In contrast, the sensitivity and specificity were particularly low in the CA9-positive SCC cases (36% and 69%, respectively).

**Conclusion:**

[^18^F]FDG PET/CT might not be useful for diagnosing lymph-node metastases of CA9-positive SCC cases of NSCLC.

## Introduction

Lung cancer is the leading cause of the global cancer incidence and mortality, accounting for an estimated 2 million diagnoses and 1.8 million deaths per year [1]. About 85% of lung cancers are non-small cell lung cancer (NSCLC), comprising 40% adenocarcinoma, 25%–30% squamous cell carcinoma (SCC), and 10%–15% large cell carcinoma [2]. Surgical resection is the standard treatment for patients with stage I or stage II NSCLC. Neoadjuvant chemotherapy or chemoradiotherapy is recommended for stage IIIA patients, and pharmacotherapy is recommended for patients at stage IIIB. Since lymph-node metastasis is one of the key factors in the determination of a patient’s clinical stage, it is important to obtain an accurate diagnosis of lymph-node metastasis before surgery in order to select the therapeutic strategy for NSCLC patients [3].

[^18^F] Fluorodeoxyglucose positron emission tomography/computed tomography ([^18^F]FDG PET/CT) is a useful tool for the diagnosis of lymph-node metastasis [4]. [^18^F]FDG PET/CT imaging reflects the increasing glucose metabolism in cancer cells compared to that of normal cells. The activated glucose metabolism results in an elevated uptake of [^18^F]FDG PET/CT values. The value of SUV accumulation is used as an indicator to determine the malignancy of tumor or presence of lymph node metastasis [5].

The transmembrane enzyme carbonic anhydrase 9 (CA9) plays a role in the regulation of the body’s pH through the reversible hydration of carbon dioxide to carbonic acid. It has been reported that the expression of CA9 in tumor cells indicates the presence of a hypoxic tumor environment [6, 7]. Due to the Warburg effect, cancer tissue frequently creates a hypoxic and acidic tumor environment compared to the normal tissue environment [8, 9]. A close correlation between [^18^F]FDG PET/CT accumulation in cancer tissue and CA9 expression has been reported [10], and a correlation between CA9 expression and lymph-node metastasis in breast and bladder cancer was described [11, 12]. There appears to be no published report of a correlation between CA9 expression and lymph-node metastasis in NSCLC, however. In this study, the correlation between the CA9 expression for lymph-node metastases in NSCLC and [^18^F]FDG PET/CT results was investigated in order to clarify the efficacy of [^18^F]FDG PET/CT for detecting lymph-node metastases of NSCLC patients.

## Patients and methods

### Patients and clinicopathologic background

Among the 564 patients who underwent primary surgical treatment for NSCLC between 2010 and 2016 at our hospital, a total of 338 patients who underwent preoperative [^18^F]FDG PET/CT were included in this study. Pathological findings were determined according to 8th edition of the Union for International Cancer Control TNM classification. Retrospective observations were conducted, with a data cutoff date of July 31, 2023, spanning a maximum of 10 years. At the cut-off date or 10 years after the start of observation, cases with no events were terminated from observation. Medical records were accessed between August 1, 2023, and September 30, 2023, for the collection of information, including personally identifiable details, related to registered patients. The study was approved by the Osaka Metropolitan University Ethics Committee (ref. no. 2019–006) and conducted in accord with the Declaration of Helsinki (as revised in 2013). Informed consent was obtained from all patients in written form. All methods that could affect the patients were performed in accord with the relevant guidelines and regulations. A lymph node with maximum standardized uptake value (SUVmax) ≥2.5 on [^18^F]FDG PET/CT was preoperatively defined as metastatic lymph nodes. And we did not distinguish whether the metastatic lymph nodes were hilar or mediastinal.

### [^18^F]FDG-PET/CT imaging

Patients underwent [^18^F]FDG-PET/CT scanning using a Biograph 16 scanner (Siemens Medical Solutions, Erlangen, Germany). The Biograph 16 consists of a 16-slice CT detector and a Lu2SiO5[Ce] crystal block, which were periodically inspected four times a year. The PET scanner field of view had an 830-mm ring diameter and a 162-mm axial length. The patient was fasting for 6 h prior to the examination. The injected activity was 3 MBq/kg of [^18^F]FDG, and PET scans were acquired from the parietal to the proximal thigh at 2 min on bed position, 60 min after injection. PET images were reconstructed using iterative reconstruction (iterations: 2, subsets: 8). Noise reduction was performed by smoothing the images with a Gaussian filter (full width at half maximum, 5 mm). Whole-body CT scans were acquired with patients in the supine position, from the parietal to the proximal thigh (120 kVp; 100 mA in auto mA mode). All data were subjected to attenuation correction based on CT data. The scatter correction followed a single-scatter simulation method [13].

### SUV max measurement

We analyze the diagnostic performance of [^18^F]FDG-PET/CT on a patient basis and the SUVmax of [^18^F]FDG-PET/CT at the lymph node was measured in the axial image slice with the highest [^18^F]FDG activity concentration. The SUVmax was calculated as follows: SUVmax = maximum radioactivity concentration in tissue (Bq/g)/[injected dose (Bq)/patient’s body weight (g)]. For patients with multiple lymph node metastases, the SUVmax of the largest lymph node was used for statistical analysis.

### Immunohistochemical staining of CA9

Paraffin-embedded sections of primary tumor specimens from all 338 patients and biopsy specimens of 48 patients underwent deparaffinization in xylene followed by hydration in descending concentrations of ethyl alcohol. The sections were then treated with 3% hydrogen peroxide to inhibit endogenous peroxidase activity, followed by a 10-min heat treatment at 105°C in an autoclave using Target Retrieval Solution (Dako, Carpinteria, CA, USA). Nonspecific binding was blocked by incubation with 10% normal rabbit serum for 10 min.

**Fig 1:**
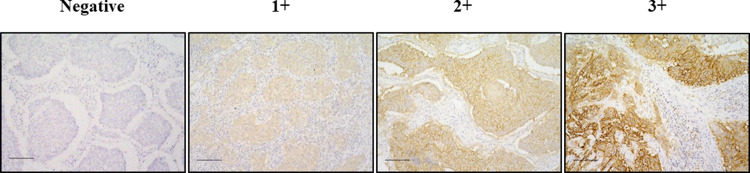
Representative images of immunostaining intensity of CA9 expression in patients with NSCLC. CA9 expression was mainly expressed at cancer cells. Intensity score was determined as follows. 0, negative; 1+, weakly positive; 2+, positive; 3+, strongly positive. Bar, 100 μm.

The specimens were then incubated with CA9 antibody (NB100–417; 1:1000; Novus Biologicals, Centennial, CO; RRID: AB_10003398) for 30 min at room temperature. These sections were incubated with a mouse linker for 10 min, a peroxidase-labeled polymer (Histofine SAB-PO(M) kit, Nichirei Biosciences, Tokyo) for 5 min, and then counterstaining with Mayer’s hematoxylin. Tumor cells’ membranes are stained by CA9, and in this study the CA9 immunoreactivity was assessed based on the intensity of membranous staining at the tumor’s deepest level and the percentage of immunoreactive cells. The immunostaining intensity score was rated from 0 to 3 as follows: 0, negative; 1+, weakly positive; 2+, positive; and 3+, strongly positive (**Fig 1**).

The immunostaining proportion score was measured as an estimate of the proportion of positive cells: 0, no immunoreactive cells; 1+, <40% immunoreactive cells; 2+, 40%–70% immunoreactive cells; and 3+, >70% immunoreactive cells. The total score was calculated as the sum of the immunostaining intensity score and proportion score ranging from 0 to 6, and CA9 expression was considered positive when the summation score was ≥4.

### Statistical analyses

The χ^2^-test was used to determine the significance of differences between covariates. Mann-Whitney U-test was used to comparisons between the groups. Survival durations were calculated using the Kaplan-Meier method and analyzed using the log-rank test to compare the cumulative survival durations in the patient groups. The Cox proportional hazards model was used to determine the multivariable hazard ratios associated with the study parameters. For all analyses, statistical significance was defined as a p-value <0.05. All statistical analyses were performed with EZR (Saitama Medical Center, Jichi Medical University, Saitama, Japan), which is a graphical user interface of R, and more precisely, a modified version of the R commander (The R Foundation for Statistical Computing, Vienna, Austria) [14].

## Results

### Association between CA9 expression and clinicopathological background

**Fig 2:**
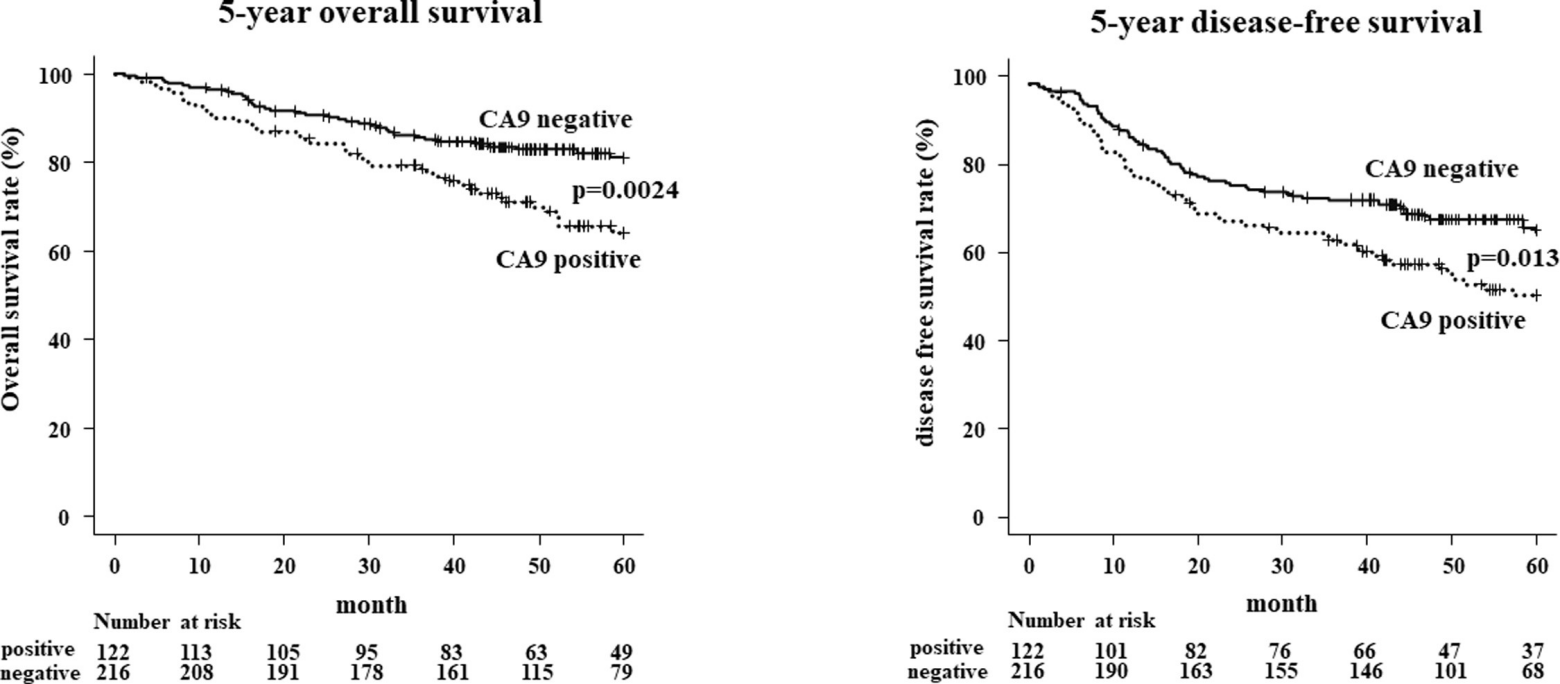
Overall survival and disease-free survival in according to CA9 expression. Patients showing positive CA9 expression have significantly poorer survival rates for OS (*p* = 0.0024) than those showing negative CA9 expression rate in 338 patients with NSCLC. Patients showing positive CA9 expression have significantly poorer survival rates for DFS (*p* = 0.013) than those showing negative CA9 expression.

The association between CA9 expression and the clinicopathologic background of the 338 patients with NSCLC is shown in **Table 1**. CA9 positivity was detected in 122 patients; the other 216 patients were CA9-negative. The CA9-positive NSCLC cases were more likely to progress to pleural invasion, pT-factor, pN-factor and pStage compared to the CA9-negative NSCLC cases. The 5-year overall survival rate (OS) of the CA9-positive patients was significantly poorer than that of the CA9-negative patients (*p* = 0.0024). The 5-year disease-free survival rate (DFS) of the CA9-positive patients was also significantly poorer than that of the CA9-negative patients (*p* = 0.013) (**Fig 2**). The results of univariate and multivariable analyses of the patients’ prognoses are shown in **Table 2**. In the univariate analysis, the histological type, lymphatic invasion, pleural invasion, T-factor, N-factor, and CA9 positivity were prognostic factors for OS. In the multivariable analysis, histological SCC and CA9 positivity were independent poor-prognosis factors for OS. The expression of CA9 in primary tumors of NSCLC may thus be a valuable independent prognostic factor for OS.

**Table 1:** Relationship between expression of CA9 and clinicopathological features in 338 patients with NSCLC.

		CA9 expression		
Variables		Positive (N = 122)	Negative (N = 216)	P value
**Age**	<65 ≧65	31(25.4%) 91(74.6%)	57(26.4%) 159(73.6%)	0.898
**Sex**	Female Male	36(29.5%) 86(70.5%)	78(36.1%) 138(63.9%)	0.233
**Smoking**	Negative Positive	28(23.3%) 92(76.7%)	50(23.4%) 164(76.6%)	1
**Pathology**	Adenocarcinoma Squamous cell carcinoma Others	80(65.6%) 37(30.3%) 5(4.1%)	144(66.7%) 55(25.4%) 17(7.9%)	0.308
**Lymphatic invasion**	Negative Positive	82(67.2%) 40(32.8%)	153(70.8%) 63(29.2%)	0.539
**Venous invasion**	Negative Positive	90(73.8%) 32(26.2%)	175(81.0%) 41(19.0%)	0.131
**Pleural invasion**	Negative Positive	74(60.7%) 48(39.3%)	163(75.5%) 53(24.5%)	**0.00631**
**Pathological T factor**	T1/2 T3/4	90(73.8%) 32(26.2%)	186(86.1%) 30(13.9%)	**0.00802**
**Pathological N factor**	N0 N1/2/3	84(68.9%) 38(31.1%)	171(79.2%) 45(20.8%)	**0.0364**
**Pathological Stage**	Ⅰ/Ⅱ Ⅲ/Ⅳ	87(71.3%) 35(28.7%)	175(81.0%) 41(19.0%)	**0.043**

**Table 2:** Univariate and multivariable analysis for 5-year overall survival of 338 patients with NSCLC.

	Univariate analysis			Multivariable analysis		
Variables	HR	95% CI	P value	HR	95% CI	P value
Age(≧65 vs<65)	1.18	0.70–1.96	0.541			
Smoking history (Yes vs No)	1.64	0.88–3.04	0.116	1.68	0.89–3.15	0.11
Histology (Sq vs non Sq)	1.84	1.16–2.92	**0.0094**	1.72	1.07–2.78	**0.025**
Lymphatic invasion (ly1 vs ly0)	1.92	1.23–3.02	**0.005**			
Vascular invasion (v1/2 vs v0)	1.36	0.81–2.28	0.25			
Pleural invasion (pl1/2/3 vs pl0)	1.70	1.07–2.70	**0.025**	1.38	0.83–2.29	0.22
T factor (T3/4 vs T1/2)	2.20	1.34–3.62	**0.0018**	1.43	0.82–2.48	0.21
N factor (N1/2/3 vs N0)	1.80	1.12–2.88	**0.015**	1.37	0.84–2.25	0.21
CA9 expression (positive vs negative)	1.97	1.26–3.09	**0.0029**	1.69	1.07–2.67	**0.025**

### Association between CA9 expression and the diagnosis of lymph-node metastasis using [^18^F]FDG PET/CT

The association between FDG accumulation in lymph nodes on [^18^F]FDG PET/CT and pathological N-factors is shown in **Table 3**. In the CA9-negative group, there was a significant association between pathological lymph-node metastasis and [^18^F]FDG PET/CT accumulation in all 216 patients. In the group of 122 patients who were CA9-positive, a significant association was also revealed between pathological lymph-node metastasis and [^18^F]FDG PET/CT accumulation. However, in the cases with SCC, no significant association was observed between pathological lymph-node metastasis and [^18^F]FDG PET/CT accumulation. The CA9-positive SCC cases exhibited a higher prevalence of false-positive or false-negative results.

**Table 3:** Comparison of lymph node accumulation on [^18^F]FDG PET/CT and pathological lymph node metastasis.

	PET LN				PET LN		
CA9 positive(n = 122)	−	+	P value	CA9 negative(n = 216)	−	+	P value
All cases				All cases			
pN0	68	16		pN0	159	12	
pN+	16	22	<0.001	pN+	22	23	<0.001
Ad enocarcinoma				Adenocarcinoma			
pN0	47	7		pN0	112	5	
pN+	8	18	<0.001	pN+	15	12	<0.001
Squamous cell carcinoma				Squamous cell carcinoma			
pN0	18	8		pN0	38	4	
pN+	7	4	1	pN+	5	8	<0.001

**Table 4** presents the sensitivity and specificity data of [^18^F]FDG PET/CT for detecting lymph-node metastasis. For the total patient population, the sensitivity and specificity of [^18^F]FDG PET/CT for lymph-node metastases were 54% and 89%, respectively. In contrast, the sensitivity and specificity for lymph-node metastases in the SCC group were low at 50% and 82%, respectively. The sensitivity and specificity were particularly low in the CA9-positive SCC cases (36% and 69%, respectively). The CA9-positive SCC cases had lower sensitivity and specificity compared to the other cases.

**Table 4:** Sensitivity and specificity of lymph nodes metastasis based on [^18^F]FDG PET/CT.

	Sensitivity (95% CI)	Specificity (95% CI)
All cases	54% (0.43–0.65)	89% (0.85–0.93)
CA9 positive	58% (0.41–0.74)	81% (0.71–0.89)
CA9 negative	51% (0.36–0.66)	93% (0.88–0.96)
Adenocarcinoma	57% (0.42–0.70)	93% (0.88–0.96)
CA9 positive	69% (0.48–0.86)	87% (0.75–0.95)
CA9 negative	44% (0.26–0.65)	96% (0.90–0.99)
Squamous cell carcinoma	50% (0.29–0.70)	82% (0.71–0.91)
CA9 positive	36% (0.11–0.69)	69% (0.48–0.86)
CA9 negative	62% (0.32–0.86)	90% (0.77–0.97)

### Association of CA9 expression between biopsy sample s and surgical specimens

We performed additional experiments of CA9 immunostaining of 48 biopsy samples from the 338 lung cancer patients, because we could obtain the 48 biopsy specimens in our hospital and confirm to be histologically carcinoma. **Table 5.** presents the association of CA9 expression between biopsy samples and surgical specimens. CA9 expression of the biopsy samples was significantly (*p*<0.01; χ^2^ test) associated with CA9 expression of surgical specimens. These findings indicated that the CA9 expression of biopsy sample might help to predict the diagnostic performance of FDG-PET imaging in lung cancer.

**Table 5:** Association of CA9 expression between biopsy samples and surgical specimens.

	Surgical specimens		
	CA9 +	CA9 −	P value
Biopsy samples			
CA9 +	16	5	
CA9 −	8	19	<0.01

**Fig 3:**
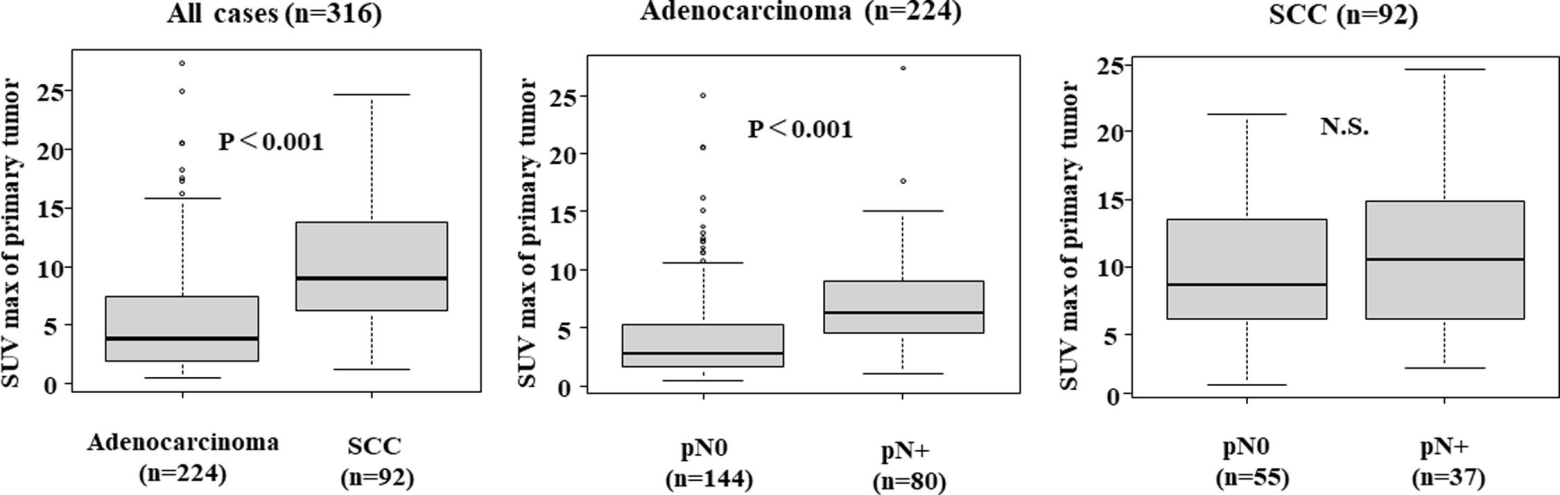
Differences in SUVmax values of primary tumors based on histological type. SUVmax value of the primary tumor was significantly higher (*p*<**0.001**) in SCC in compared to adenocarcinoma. Adenocarcinoma patients with lymph node metastases had significantly higher SUVmax values of the primary tumor than those without lymph node metastases. In contrast, no significant difference of SUVmax value was found between SCC patients with lymph node metastases and those without lymph node metastases.

**Fig 3** depicts the differences in the SUVmax values of the primary tumor according to histological type. The SUVmax values of the SCC cases was significantly high in the primary tumors compared to adenocarcinoma. In addition, the SUVmax values of the adenocarcinomas were significantly higher in the cases with lymph-node metastasis, whereas there was no significant difference between the lymph-node metastasis and SUVmax values of the SCCs.

## Discussion

The present results demonstrated that the expression of CA9 in primary tumors was associated with pleural invasion and the pathological T-factor, N-factor, and clinical stage in NSCLC. CA9 is considered a marker of hypoxia and is activated when hypoxia-inducible factor 1-alpha (HIF-1α) accumulates [15], and hypoxia has been reported to be associated with increased tumor cell viability and malignancy and to promote tumor cell growth [16, 17]. It has also reported that CA9 is associated with tumor invasion and metastasis [18, 19]. These reports suggest that CA9-positive cases tend to develop to an advanced stage. As a result, in the present study positive CA9 expression in the primary tumor was an independent poor prognostic factor for OS in a multivariable analysis.

The histological type of SCC was also an independent poor prognostic factor for OS in the multivariable analysis. SCC is associated with smoking and is more common in men than women. The prognosis for NSCLC is also significantly lower in men than in women, as men have a shorter life expectancy than women [20]. It has been reported that deaths from diseases other than lung cancer such as ischemic heart disease, stroke, and interstitial pneumonia were significantly more common in SCC compared to adenocarcinoma, because SCC is associated with smoking [21–23]. As a result, the histological type of SCC was an independent poor prognostic factor for OS in the present multivariable analysis.

Curative surgical resection is important for patients with NSCLC. An accurate diagnosis of lymph-node metastasis before surgery is important in the selection of the surgical procedure for NSCLC patients. [^18^F]FDG PET/CT is a useful and dependable tool to diagnose lymph-node metastasis of lung cancer. The reported sensitivity and specificity of [^18^F]FDG PET/CT for identifying lymph-node metastasis in a series of NSCLC patients were 68% and 93%, respectively [24]. In the present investigation, the respective sensitivity and specificity values of [^18^F]FDG PET/CT for detecting lymph-node metastasis were 57% and 93% in 224 adenocarcinomas but low at only 36% and 69% in the CA9-positive SCC cases. It has been reported that the sensitivity and specificity of [^18^F]FDG PET/CT for identifying lymph-node metastasis were relatively low in SCC compared to adenocarcinoma [25–27]. Because SCC is associated with tobacco smoking, which frequently results in inflammatory diseases such as COPD and interstitial pneumonia [28], SCC patients with inflammatory lung diseases may accumulate high [^18^F]FDG in their non-metastatic lung lymph nodes because inflammatory cells such as macrophages consume large amounts of glucose. [29–32]. Therefore, SCC with inflammatory lung diseases is frequently recognized as false-positive lymph-node metastasis by [^18^F]FDG PET/CT, which might result in the low sensitivity of [^18^F]FDG PET/CT for lymph-node metastasis. In addition, false-positive lymph-node metastasis suggested by [^18^F]FDG PET/CT was more common especially in CA9-positive SCC cases. Inflammation has been reported to frequently induce hypoxia as determined by CA9-positivity [33]. Compared to adenocarcinoma, SCC is a more hypoxic environment [34]. So, in CA9-positive group of SCC, CA9 failed to reflect tumor hypoxia because of frequent hypoxic lesions caused by inflammation. On the other hand, in adenocarcinoma, CA9 accurately reflected tumor hypoxia, which may explain the increased sensitivity of [^18^F]FDG PET/CT in the CA9-positive group of adenocarcinoma. These data may indicate that [^18^F]FDG PET/CT is not a useful tool to identify lymph-node metastases in CA9-positive SCC cases of NSCLC.

SCC has been reported to have a higher SUV of the primary tumor compared to adenocarcinoma [20, 25], and a higher SUV of the primary tumor is correlated with a higher likelihood of occult nodal metastasis [35–37], which might be one of the reasons why the CA9-positive SCC cases in the present study had more false-negative outcomes. Fig 3 indicated that in adenocarcinoma, patients with lymph node metastasis had significantly higher SUV values of the primary tumor, while in SCC, there was no association between lymph node metastasis and SUV values of primary tumor, suggesting that in adenocarcinoma, the SUV values of primary tumor could predict lymph node metastasis but not in SCC. Based on these results, [^18^F]FDG PET/CT is not a useful tool for predicting lymph node metastasis in CA9-positive SCC. So CA9-positive SCC would require not only [^18^F]FDG PET/CT but also other assessments such as MRI and endobronchial ultrasound-guided transbronchial needle aspiration (EBUS-TBNA) to diagnose lymph-node metastasis pre-operatively.

This study has several limitations. First, the results of this study were from a single institution, so most patients are a single race and sample size is small. Second, we included patients only who underwent surgical treatment, so patients who didn’t undergo surgery were excluded. Therefore, the results may be different from the observed result if all patients diagnosed with lung cancer were included. Also, SUV max ≥ 2.5 is considered positive in this study, so the results may be different from the observed results depending on the setting. Third, its design was retrospective, not all factors could be examined in all patients, and some cases were lost during the follow-up. In addition, CA9 was examined only by immunostaining, and it would have been more clinically feasible if not only the protein level but also the mRNA level could have been examined because mRNA analysis has the advantage of providing quantified values, allowing for a more accurate evaluation.

## Conclusion

[^18^F]FDG PET/CT might not be useful for diagnosing lymph-node metastases of CA9-positive SCC cases of NSCLC.

## Supporting information

S1 Data (XLSX)

## References

[1] Thandra KC, Barsouk A, Saginala K, Aluru JS, Barsouk A. Epidemiology of lung cancer. Contemp Oncol (Pozn). 2021;25(1):45–52. doi: 10.5114/wo.2021.103829 33911981 PMC8063897

[2] Schabath MB, Cote ML. Cancer Progress and Priorities: Lung Cancer. Cancer Epidemiol Biomarkers Prev. 2019;28(10):1563–79. doi: 10.1158/1055-9965.EPI-19-0221 31575553 PMC6777859

[3] Zhang C, Pang G, Ma C, Wu J, Wang P, Wang K. Preoperative Risk Assessment of Lymph Node Metastasis in cT1 Lung Cancer: A Retrospective Study from Eastern China. J Immunol Res. 2019;2019:6263249. doi: 10.1155/2019/6263249 31886306 PMC6914921

[4] Heiden BT, Chen G, Hermann M, Brown RKJ, Orringer MB, Lin J, et al. 18F-FDG PET intensity correlates with a hypoxic gene signature and other oncogenic abnormalities in operable non-small cell lung cancer. PLoS One. 2018;13(7):e0199970. doi: 10.1371/journal.pone.0199970 29966011 PMC6028077

[5] Zhao L, He ZY, Zhong XN, Cui ML. (18)FDG-PET/CT for detection of mediastinal nodal metastasis in non-small cell lung cancer: a meta-analysis. Surg Oncol. 2012;21(3):230–6. doi: 10.1016/j.suronc.2011.11.001 22197027

[6] Kim SJ, Rabbani ZN, Vollmer RT, Schreiber EG, Oosterwijk E, Dewhirst MW, et al. Carbonic anhydrase IX in early-stage non-small cell lung cancer. Clin Cancer Res. 2004;10(23):7925–33. doi: 10.1158/1078-0432.CCR-04-0636 15585626

[7] Mees G, Vangestel C, Dierckx R, Pauwels P, Van Meerbeeck J, Van de Wiele C. Carbonic anhydrase IX expression correlates with FDG uptake by primary non-small cell lung cancer. Cancer Biother Radiopharm. 2010;25(2):149–54. doi: 10.1089/cbr.2009.0658 20423227

[8] Warburg O. On respiratory impairment in cancer cells. Science. 1956;124(3215):269–70. 13351639

[9] Schwartz L, Supuran CT, Alfarouk KO. The Warburg Effect and the Hallmarks of Cancer. Anticancer Agents Med Chem. 2017;17(2):164–70. doi: 10.2174/1871520616666161031143301 27804847

[10] Han MW, Lee HJ, Cho KJ, Kim JS, Roh JL, Choi SH, et al. Role of FDG-PET as a biological marker for predicting the hypoxic status of tongue cancer. Head Neck. 2012;34(10):1395–402. doi: 10.1002/hed.21945 22052623

[11] Eom KY, Jang MH, Park SY, Kang EY, Kim SW, Kim JH, et al. The Expression of Carbonic Anhydrase (CA) IX/XII and Lymph Node Metastasis in Early Breast Cancer. Cancer Res Treat. 2016;48(1):125–32. doi: 10.4143/crt.2014.243 25761481 PMC4720081

[12] Ord JJ, Agrawal S, Thamboo TP, Roberts I, Campo L, Turley H, et al. An investigation into the prognostic significance of necrosis and hypoxia in high grade and invasive bladder cancer. J Urol. 2007;178(2):677–82. doi: 10.1016/j.juro.2007.03.112 17574616

[13] Ito R, Yashiro M, Tsukioka T, Izumi N, Komatsu H, Inoue H, et al. Usefulness of pyruvate dehydrogenase-E1α expression to determine SUVmax cut-off value of [(18)F]FDG-PET for predicting lymph node metastasis in lung cancer. Sci Rep. 2023;13(1):1565. 36709375 10.1038/s41598-023-28805-8 PMC9884208

[14] Kanda Y. Investigation of the freely available easy-to-use software ’EZR’ for medical statistics. Bone Marrow Transplant. 2013;48(3):452–8. doi: 10.1038/bmt.2012.244 23208313 PMC3590441

[15] Supuran CT. Carbonic Anhydrase Inhibition and the Management of Hypoxic Tumors. Metabolites. 2017;7(3). doi: 10.3390/metabo7030048 28926956 PMC5618333

[16] Semenza GL. Targeting HIF-1 for cancer therapy. Nat Rev Cancer. 2003;3(10):721–32. doi: 10.1038/nrc1187 13130303

[17] Hockel M, Schlenger K, Aral B, Mitze M, Schaffer U, Vaupel P. Association between tumor hypoxia and malignant progression in advanced cancer of the uterine cervix. Cancer Res. 1996;56(19):4509–15. 8813149

[18] Cairns RA, Hill RP. Acute hypoxia enhances spontaneous lymph node metastasis in an orthotopic murine model of human cervical carcinoma. Cancer Res. 2004;64(6):2054–61. doi: 10.1158/0008-5472.can-03-3196 15026343

[19] Shin HJ, Rho SB, Jung DC, Han IO, Oh ES, Kim JY. Carbonic anhydrase IX (CA9) modulates tumor-associated cell migration and invasion. J Cell Sci. 2011;124(Pt 7):1077–87. doi: 10.1242/jcs.072207 21363891

[20] Nakamura H, Sakai H, Kimura H, Miyazawa T, Marushima H, Saji H. Difference in Postsurgical Prognostic Factors between Lung Adenocarcinoma and Squamous Cell Carcinoma. Ann Thorac Cardiovasc Surg. 2017;23(6):291–7. doi: 10.5761/atcs.oa.17-00020 28966230 PMC5738450

[21] Kawase A, Yoshida J, Ishii G, Nakao M, Aokage K, Hishida T, et al. Differences between squamous cell carcinoma and adenocarcinoma of the lung: are adenocarcinoma and squamous cell carcinoma prognostically equal? Jpn J Clin Oncol. 2012;42(3):189–95. doi: 10.1093/jjco/hyr188 22210923

[22] Sakurai H, Asamura H, Goya T, Eguchi K, Nakanishi Y, Sawabata N, et al. Survival differences by gender for resected non-small cell lung cancer: a retrospective analysis of 12,509 cases in a Japanese Lung Cancer Registry study. J Thorac Oncol. 2010;5(10):1594–601. doi: 10.1097/JTO.0b013e3181f1923b 20736855

[23] Hao B, Li F, Wan X, Pan S, Li D, Song C, et al. Squamous cell carcinoma predicts worse prognosis than adenocarcinoma in stage IA lung cancer patients: A population-based propensity score matching analysis. Front Surg. 2022;9:944032. doi: 10.3389/fsurg.2022.944032 36090323 PMC9461700

[24] Sun J, Li Y, Gong F, Xu S, Wu J, Wang H, et al. The diagnostic value of PET/CT for the lymph node metastasis in Asian patients with non-small cell lung cancer: A meta-analysis. Hell J Nucl Med. 2022;25(2):196–204. doi: 10.1967/s002449912479 35913866

[25] Damirov F, Stoleriu MG, Manapov F, Büsing K, Michels JD, Preissler G, et al. Histology of the Primary Tumor Correlates with False Positivity of Integrated 18F-FDG-PET/CT Lymph Node Staging in Resectable Lung Cancer Patients. Diagnostics (Basel). 2023;13(11). doi: 10.3390/diagnostics13111893 37296745 PMC10252240

[26] Wang Y, Ma S, Dong M, Yao Y, Liu K, Zhou J. Evaluation of the factors affecting the maximum standardized uptake value of metastatic lymph nodes in different histological types of non-small cell lung cancer on PET-CT. BMC Pulm Med. 2015;15:20. doi: 10.1186/s12890-015-0014-2 25880540 PMC4372315

[27] Ismail M, Nachira D, Swierzy M, Ferretti GM, Englisch JP, Ossami Saidy RR, et al. Lymph node upstaging for non-small cell lung cancer after uniportal video-assisted thoracoscopy. J Thorac Dis. 2018;10(Suppl 31):S3648–s54. doi: 10.21037/jtd.2018.06.70 30505548 PMC6258647

[28] Bozinovski S, Vlahos R, Anthony D, McQualter J, Anderson G, Irving L, et al. COPD and squamous cell lung cancer: aberrant inflammation and immunity is the common link. Br J Pharmacol. 2016;173(4):635–48. Epub doi: 10.1111/bph.13198 . 26013585 PMC4742298

[29] An YS, Sun JS, Park KJ, Hwang SC, Park KJ, Sheen SS, et al. Diagnostic performance of (18)F-FDG PET/CT for lymph node staging in patients with operable non-small-cell lung cancer and inflammatory lung disease. Lung. 2008;186(5):327–36. doi: 10.1007/s00408-008-9109-3 18670805

[30] Takamochi K, Yoshida J, Murakami K, Niho S, Ishii G, Nishimura M, et al. Pitfalls in lymph node staging with positron emission tomography in non-small cell lung cancer patients. Lung Cancer. 2005;47(2):235–42. doi: 10.1016/j.lungcan.2004.08.004 15639722

[31] Konishi J, Yamazaki K, Tsukamoto E, Tamaki N, Onodera Y, Otake T, et al. Mediastinal lymph node staging by FDG-PET in patients with non-small cell lung cancer: analysis of false-positive FDG-PET findings. Respiration. 2003;70(5):500–6. doi: 10.1159/000074207 14665776

[32] Vaidyanathan S, Patel CN, Scarsbrook AF, Chowdhury FU. FDG PET/CT in infection and inflammation—current and emerging clinical applications. Clin Radiol. 2015;70(7):787–800. doi: 10.1016/j.crad.2015.03.010 25917543

[33] Eltzschig HK, Carmeliet P. Hypoxia and inflammation. N Engl J Med. 2011;364(7):656–65. doi: 10.1056/NEJMra0910283 21323543 PMC3930928

[34] Schuurbiers OC, Meijer TW, Kaanders JH, Looijen-Salamon MG, de Geus-Oei LF, van der Drift MA, et al. Glucose metabolism in NSCLC is histology-specific and diverges the prognostic potential of 18FDG-PET for adenocarcinoma and squamous cell carcinoma. J Thorac Oncol. 2014;9(10):1485–93. doi: 10.1097/JTO.0000000000000286 25170642

[35] Zhou X, Chen R, Huang G, Liu J. Potential clinical value of PET/CT in predicting occult nodal metastasis in T1-T2N0M0 lung cancer patients staged by PET/CT. Oncotarget. 2017;8(47):82437–45. Epub doi: 10.18632/oncotarget.19535 . 29137276 PMC5669902

[36] Kim DH, Song BI, Hong CM, Jeong SY, Lee SW, Lee J, et al. Metabolic parameters using ^18^F-FDG PET/CT correlate with occult lymph node metastasis in squamous cell lung carcinoma. Eur J Nucl Med Mol Imaging. 2014;41(11):2051–7. 24990401 10.1007/s00259-014-2831-6

[37] Miyasaka Y, Suzuki K, Takamochi K, Matsunaga T, Oh S. The maximum standardized uptake value of fluorodeoxyglucose positron emission tomography of the primary tumour is a good predictor of pathological nodal involvement in clinical N0 non-small-cell lung cancer. Eur J Cardiothorac Surg. 2013;44(1):83–7. doi: 10.1093/ejcts/ezs604 23233074

